# Automatic Contrast Enhancement of Brain MR Images Using Hierarchical Correlation Histogram Analysis

**DOI:** 10.1007/s40846-015-0096-6

**Published:** 2015-11-21

**Authors:** Chiao-Min Chen, Chih-Cheng Chen, Ming-Chi Wu, Gwoboa Horng, Hsien-Chu Wu, Shih-Hua Hsueh, His-Yun Ho

**Affiliations:** Department of Computer Science and Information Engineering, National Taiwan University, Taipei, 10617 Taiwan; Department of Computer Science and Engineering, National Chung Hsing University, Taichung, 40227 Taiwan; Department of Medical Imaging, Chung Shan Medical University Hospital, Taichung, 40201 Taiwan; Department of Computer Science and Information Engineering, National Taichung University of Science and Technology, 129, Sec. 3, San-min Rd., Taichung, 40401 Taiwan, ROC

**Keywords:** Image enhancement, Correlation histogram, Magnetic resonance imaging (MRI), Parkinson’s disease

## Abstract

Parkinson’s disease is a progressive neurodegenerative disorder that has a higher probability of occurrence in middle-aged and older adults than in the young. With the use of a computer-aided diagnosis (CAD) system, abnormal cell regions can be identified, and this identification can help medical personnel to evaluate the chance of disease. This study proposes a hierarchical correlation histogram analysis based on the grayscale distribution degree of pixel intensity by constructing a correlation histogram, that can improves the adaptive contrast enhancement for specific objects. The proposed method produces significant results during contrast enhancement preprocessing and facilitates subsequent CAD processes, thereby reducing recognition time and improving accuracy. The experimental results show that the proposed method is superior to existing methods by using two estimation image quantitative methods of PSNR and average gradient values. Furthermore, the edge information pertaining to specific cells can effectively increase the accuracy of the results.

## Introduction

Parkinson’s disease (PD) is a major neurological disease that occurs mostly in individuals aged 50 years or older. It is reported that about 5–10 % of individuals 20–40 years old may have PD. In response to this widespread health problem, observations have been conducted to identify cellular atrophy in the early stages to determine the probability of PD. Examination methods include contrast techniques such as computed tomography (CT) and nuclear magnetic resonance imaging (MRI). These contrast techniques are mostly used to check for pathological changes in the brain tissue structure that occur with conditions such as Alzheimer’s disease, PD, epilepsy, schizophrenia, and multiple sclerosis [1–5]. Although medical instruments provide important information, the images produced must be interpreted by medical personnel. In a medical center or hospital, many medical images are generated, so medical personnel have a significant workload. The automatic contrast enhancement image could further improve the accuracy in preprocessing of computer-aided detection/diagnosis (CAD) systems. This study aimed to develop a fully automatic enhancement method for analyzing MRI images of the brain.

**Fig. 1:**
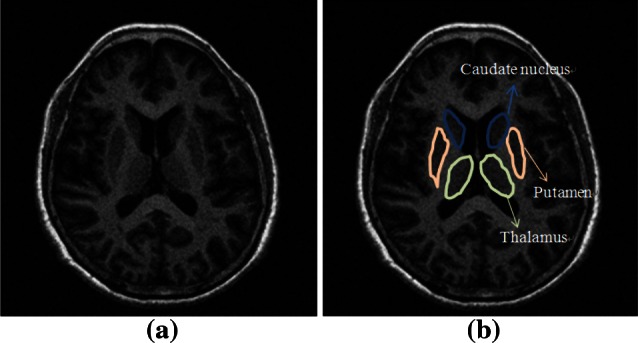
Brain MRI images of **a** normal and **b** PD-affected cells

The cardinal symptoms of PD include tremors of the hands and feet, rigidity, and postural instability. The substantial nigra area of human brain cells is related to motion control and emotional mediation. PD, which results from degeneration, pathological changes, and the death of substantial amounts of the brain’s nigra cell tissue for indeterminate reasons, reduces the number of dopaminergic neurons produced in the substantial nigra area. When more than 50 % of the substantial nigra cells are dead, the caudate nucleus, putamen, thalamus, and pallidum cells in other motion-control areas of the brain shrink accordingly. These areas eventually fail to work, leading to physical disabilities such as tremors, muscle rigidity, and slow motion. The greater the reduction of dopamine, the more serious the atrophy of the brain cells and the symptoms [6]. Many other brain diseases that can affect dopamine or other control systems, including apoplexy, hydrocephalus, and other neurodegenerative diseases, have similar symptoms in the early stages. Digital image processing technology is used to automatically enhance the contrast of brain cells with pathological changes in MRI images, so that medical professionals can diagnose these diseases (Fig. 1).

Most of the literature regarding brain image processing addresses the following three major areas [7]:

1. Extraction of the main tissues of the brain. The brain content is divided into white matter (WM), gray matter (GM), and cerebral spinal fluid (CSF) [7–9].
2. Extraction of one or more specific small tissue from, e.g., the cortex [10, 11], sulci [12, 13] and sub-cortical structures [14, 15].
3. Segmentation of anatomic cell tissues of the brain [16–18].

This study aimed at improving contrast enhancement process. Better contrast enhancement result could further provides better segmentation accuracy at the anatomical structure of the brain, and digital image processing is used to process three major atrophic cell areas of the brain affected by PD: the caudate nucleus, putamen, and thalamus (Fig. 1b). The proposed image preprocessing method can improve the PD diagnosis accuracy of CAD systems.

The rest of this study is organized as follows. Section 2 reviews studies related to image enhancement and segmentation. Section 3 describes the proposed method. Section 4 discusses the experimental results. Conclusions are given in Sect. 5.

## Literature Review

When image contrast enhancement is applied to images of brain cell tissue, the enhancement images can follow by segmentation and classification to increase these processes accuracy rates [19–22]. Common image contrast enhancement methods for segmenting brain MRI images are introduced below.

### Image Contrast Enhancement Methods

Common image contrast enhancement methods include histogram equalization (HE) [23] and contrast limited adaptive histogram equalization (CLAHE) [24]. HE accumulates the histogram of the pixel values in the image according to the imported original image and then displaces all image pixel values. It also changes the original pixel values to enhance the image contrast. In an HE-processed image, the bright area is likely to be overly intensified; as a result, the image highlight regions are overexposure, the difference in image contrast is very large, and the distribution of pixels is unnatural [25].

CLAHE uses partial histogram displacement expansion to avoid excessive contrast differences after enhancement or excessive enhancement of image noise. With CLAHE, pixels are enhanced uniformly, and the histogram range can be adjusted as required to reduce the artificiality after image enhancement [16].

Another common method is the multi-scale morphological (MSM) approach, in which the expansion and etching are operated associatively based on the concept of morphological filtering [26]. The first combination is erosion before dilation, called closing. The second combination is dilation before erosion, called opening.

Closing morphological filtering is applied for image contrast enhancement when processing the pixels in the dark region of the image. With closing morphological filtering, the pixel contrast in the shadow regions can be enhanced to effectively improve pixel information. Since the light region remains unchanged, the contrast of the enhanced image is relatively apparent and the enhanced image is relatively natural. Opening morphological filtering is used during processing of the pixels in the light region of the image. With opening morphological filtering, the contrast can be stretched in the light region of the image, and the whitening is unlikely to occur. Opening morphological filtering retains the dark details of the image and prevents abnormal expansion of dark region pixels [27].

However, in the above methods image contrast enhancement of a specific region of interest (ROI) cannot be implemented automatically. Therefore, this study applies an image correlation matrix to automate the contrast adjustment processing of ROI pixels.

### Segmentation of Brain Tissue

Pham et al. calculated the differences between the average gray-level intensity of clusters and pixels based on the principle of the fuzzy C-means (FCM) clustering method [28] to classify and segment three major tissues of the brain (GM, WM, CSF) [29]. However, this method is prone to disturbance by noise. Methods such as space constraint, topology constraint, and bias field correction are based on the FCM clustering method [8, 30–33].

Caldairou et al. proposed a segmentation method that combines a non-local (NL) framework with the FCM clustering method [7], and to processed the nonuniform intensity and noise of an MRI image. First, the robust FCM clustering method proposed by Pham et al. [8], which uses a regularization framework, is used. The method uses the clustering iterative calculation refers only to the upper, lower, left, and right pixels, to eliminate the interference of nonuniform gray-level intensity in the image. In addition, image noise is processed with the NL framework to generate weights. Therefore, the weights of clusters are changed based on their pixel values. The weights generated by the NL framework are combined with the robust FCM clustering method to remove the interference of noise and nonuniform intensity in MRI images.

Ji et al. proposed a segmentation method that combines the weighted image patch with the FCM clustering method [9]. The method corresponding weights are calculated according to the eight neighboring pixels around the center of the patch. This method, which adds weight to the segmentation of FCM iterative clustering in the local spatial information of the image, uses the patch instead of pixels as the unit, which effectively removes MRI noise interference. However, these methods are very complicated, and they cannot segment brain cells accurately.

The aforementioned segmentation methods do not use a contrast enhancement algorithm to segment brain cells, thus this study proposes an adaptive algorithm for adjusting brain cell image contrast which can improve segmentation accuracy. First, a correlation matrix is created and implemented using hierarchical analysis to improve image contrast and reduce noise in specific objects.

## Methods

**Fig. 2:**
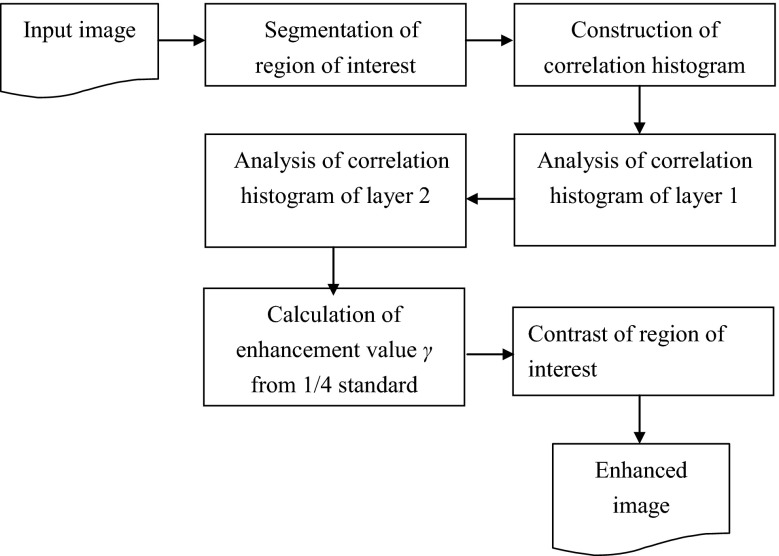
Flow chart of enhancement processes

This study proposes an adaptive algorithm for brain MRI images called the hierarchical correlation histogram analysis algorithm (HCHA). HCHA can automatically enhance three types of major PD-affected brains atrophic cells in images. This algorithm uses each object’s grayscale distribution degree of pixel intensity to construct a correlation histogram matrix from the original ROI. Then, it generates a segment of the correlation histogram matrix wherein different blocks express the correlation distribution degree of each object, since the specific objects cannot be represented in the global correlation histogram. The hierarchical analysis focuses on specific objects to represent the correlation distributions of pixels for optimization and adaptively regulates the contrast of each specific object. Figure 2 shows the overall flow chart of the proposed method.

This section introduces the process of image enhancement. The image correlation histogram is created using HCHA. An image correlation histogram for calculating space layers is proposed. The variance of the histogram is calculated according to the different correlation histograms of various layers. Finally, the variance is used to process the space contrast enhancement according to various spatial distributions. The four parts of the process are described below.

### Segmentation of ROI

**Fig. 3:**
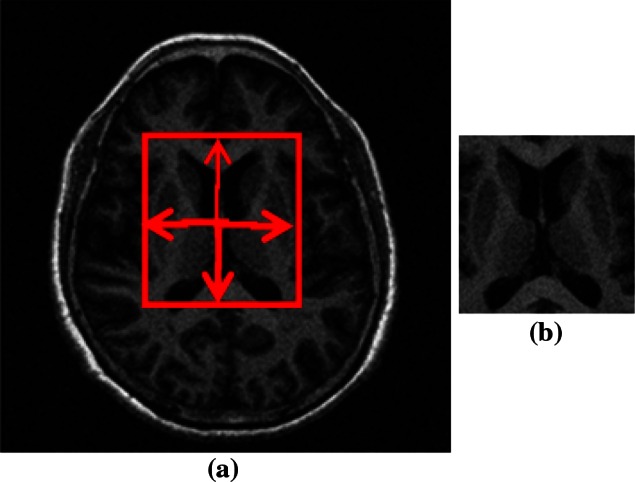
**a** Original MRI image and **b** segmented ROI

After the length and width of the original brain MRI image are calculated, an image composed of 50 pixels to the top, bottom, left, and right of the center point is extracted as the ROI, as shown in Fig. 3.

### Creation of Correlation Histogram

In the image, each specific object has a different pixel intensity distribution in the image. A 3 × 3 window is used to calculate the centroid pixel of the block between the neighboring eight pixels’ intensity distributions. If the neighboring pixels have a similar centroid pixel, then they have higher correlations with the centroid pixel. Thus, the size of the constructed correlation matrix depends on the pixels’ intensity range in the ROI. A 3 × 3 window is used to scan the overall ROI with overlapping. In Eq. (1), *cen* is the centroid pixel and *n*_*1*_*, n*_*2*_,…, *n*_*8*_ are the eight neighboring pixel values of *cen*. The correlation between *n*_*1*_*, n*_*2*_*,…, n*_*8*_ and *m* are created and marked as 1 in the corresponding coordinate position of the correlation histogram; otherwise, *m* is marked as 0 and accumulated continuously. The correlation histogram is defined as:

1 $$CorreHis = \sum\limits_{i = 1}^{x} {\sum\limits_{j = 1}^{y} {CorreHis\left( {ROI\left( {win_{m} \left( {cen} \right)} \right)} \right),ROI\left( {win{}_{m}\left( {n_{k} } \right)} \right)} } + 1))$$

**Fig. 4:**
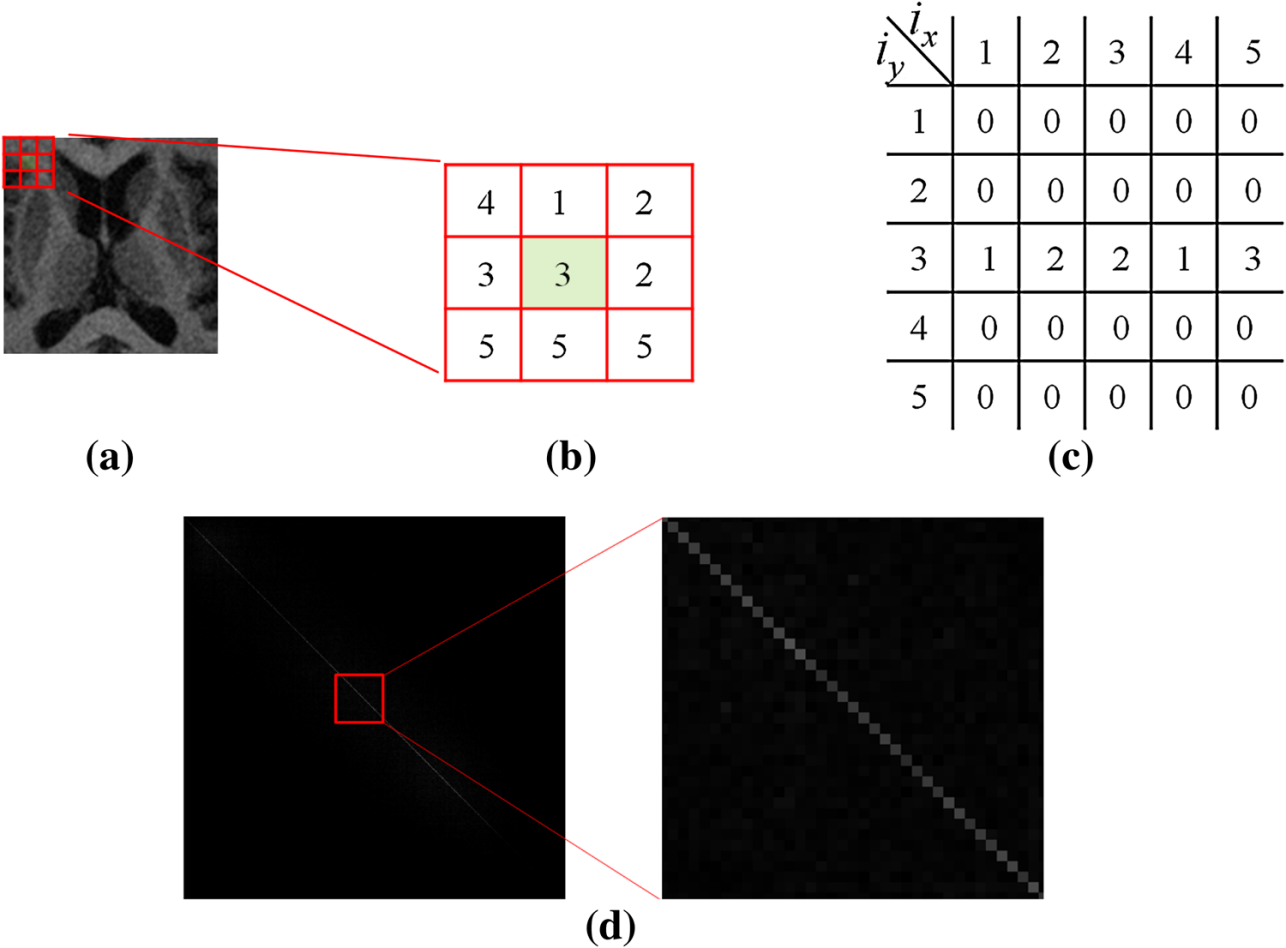
**a** Original ROI, **b** 3 × 3 ROI pixels in a block, **c** correlation histogram, and **d** result of applying correlation histogram matrix

For example, Fig. 4a shows an ROI of the original image, and Fig. 4b shows the 3 × 3 window of the ROI pixels in a block. The 3 × 3 window is used to overlap the scanning of all of the pixels in the ROI. In the window, there are nine pixels, each of which is used as the centroid point of the window. If the full image is scanned, each pixel is taken as the centroid point *cen*, and its eight neighbor points (.) are considered, as long as the pixels are identity and are marked 1 on the correlation histogram. Otherwise, 0 accumulates continuously until all pixels of the ROI are scanned. The correlation histogram of the full image is shown in Fig. 4c, where *i*_*x*_ and *i*_*y*_ are the grayscale intensity ranges.

### Analysis of Correlation Histogram

In the original ROI (Fig. 4a) correlation histogram matrix, shown in Fig. 4d, larger values such as bright pixels represent a higher correlation of grayscale pixel intensity in the 3 × 3 block. Therefore, the correlation histogram can be used to segment blocks and provide individual analysis of two distinct and relevant spatial layers.

**Fig. 5:**
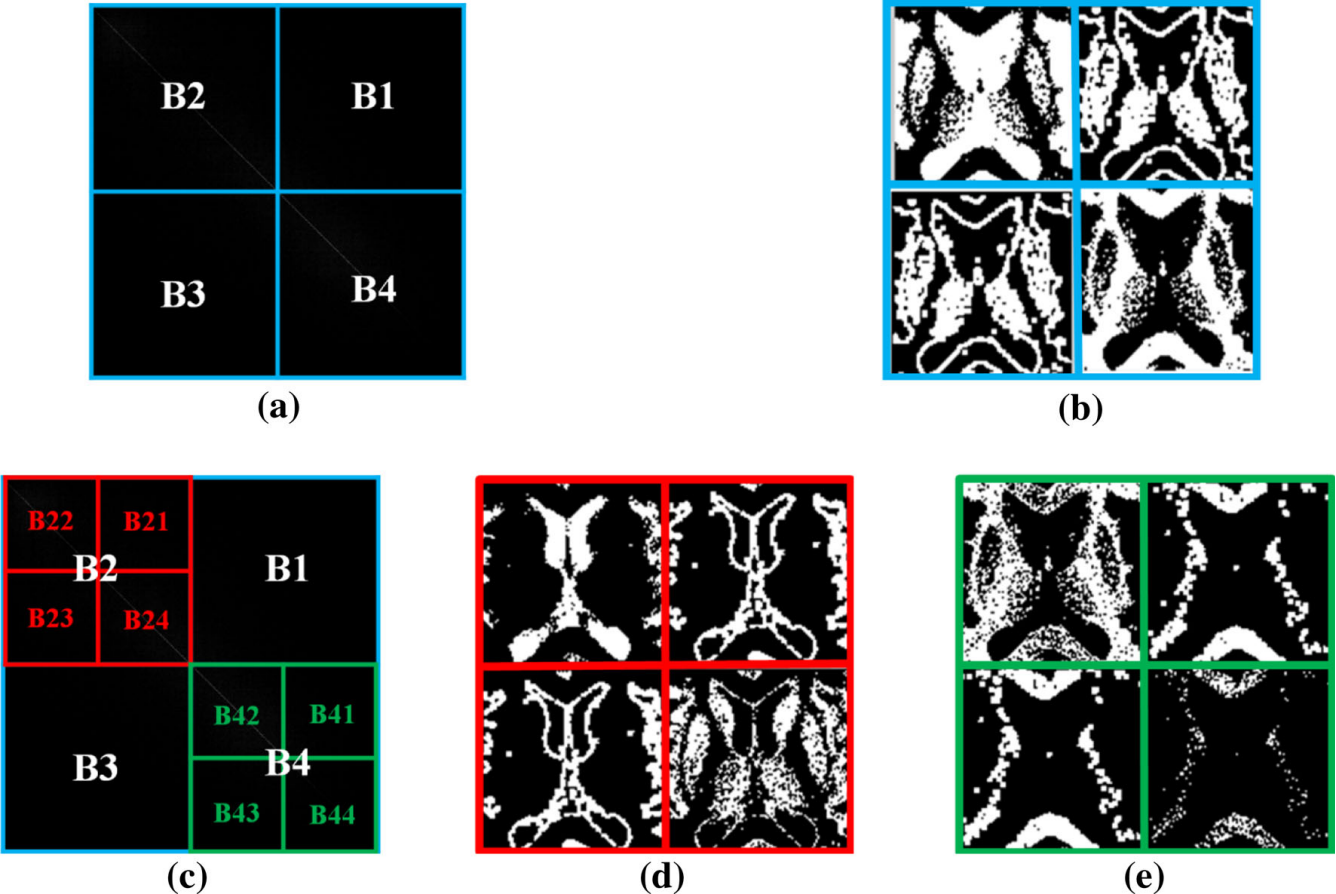
Correlation histogram block analysis. **a** Layer 1 analysis, **b** pixel distribution corresponding to layer 1, **c** layer 2 analysis, and **d**, **e** pixel distributions corresponding to layer 2

First, layer 1 is divided into four blocks, B1, B2, B3, and B4, as shown in Fig. 5a. Blocks B1 and B3, corresponding to the spatial pixel intensity of the original ROI image, represent the edge information, and blocks B2 and B4 represent the object information. Second, blocks B2 and B4 have the mapping pixel value of the original ROI. If the values are equal to 0, they represent the background and mapping pixel value of the located position from the original ROI within the assigned binary value 0. If the values are greater than 0, they represent the specific objects and mapping pixel value of the ROI-located position within the assigned binary value 1. The *B*_*m*_ of the binary images is described as Eq. (2),  where the *LocationMap*(*x*) is x-axis of values of correlation histogram  and the *LocationMap*(*y*) is y-axis of values of correlation histogram. The result of the layer 1 process is shown in Fig. 5b.

2 $$B_{m} = \left\{\begin{array}{ll} 1 , & {\text{if }}ROI (i , j ) == LocationMap(x){\text{ and }}ROI (i , j ) == LocationMap(y); \\ 0 , & {\text{otherwise}}. \\ \end{array} \right.,\;Bm = B1,B2,B3 \ldots B44$$

Because this method is used to enhance the brain cell region in the brain MRI image, B2 and B4 are segmented into four blocks for analysis. Figure 5d, e show the corresponding pixel distributions in layer 2, marked B21, B22, B23, and B24 and B41, B42, B43, and B44, as shown in Fig. 5c. They correspond to the information pertaining to the original pixel space Eq. (2). If the correlation histogram of layer 1 is used, then that obtained the conspicuous non-object information. But the information pertaining to the individual object cannot be obtained. The correlation histogram analysis of layer 2 can conspicuous the object information pertaining to individual objects, so the information can be used to adjust the pixel contrast of different objects, as shown in Figs. 5d, e.

### Calculation of Contrast Enhancement Parameter

After the correlation histogram analysis, the blocks marked B22, B21, and B24 and those marked B42, B41, and B44 extracted related to the pixel space of the original ROI image for image enhancement. First, the standard deviation σ of the ROI image is calculated as shown in Eqs. (3) and (4), where *RN* is the number of pixels of the ROI, *PV* is the value of each pixel of the ROI, and *μ* is the mean value of the ROI, representing the global standard deviation of the ROI image. The standard deviation reflects the degree of pixel dispersion and represents more objective expression between pixels that are different from the global image. With the proposed method, which segments the four blocks by calculating the correlation histogram of the ROI, computing a quarter of the standard deviation from the ROI, and focusing each block to adjust pixel values, the result of contrast enhancement is improved and appropriate. The enhancement parameter γ is calculated using Eq. (5).

3 $$\sigma = \sqrt {\frac{1}{RN}\sum\limits_{i = 1}^{N} {\left( {PV_{i} - \mu } \right)^{2} } }$$

4 $$\mu = \frac{1}{RN}\sum\limits_{i = 1}^{RN} {\left( {PV_{i} } \right)}$$

5 $$\gamma = \frac{1}{4}\sigma$$

This algorithm is segmented into four blocks from each layer for analysis of the pixels’ intensity correlation in ROI. The other important reason for the segmentation is that using the standard deviation of ROI can greatly increase the contrast amplitude and reduce the information pertaining to specific objects, with the result that the enhanced images become too bright. Otherwise, using less than 1/4 of the standard deviation is used, the contrast amplitude in the enhanced images will be insignificant. Therefore, in this process, 1/4 of the standard deviation is used to retain the information pertaining to specific objects and to more effectively achieve enhancement.

Blocks B22 and B21 reflect non-object features thus use contrast enhancement parameter γ to reduce pixels intensity. Blocks B24, B42, B41, and B44 reflect object features. The contrast-enhancement parameters of these blocks are increased. Finally, the amplitude of pixel enhancement is adjusted for all pixel values in the pixel space corresponding to blocks B22, B21, and B24 and B42, B41, and B44 to blocks. For the three subblocks in B2, the pixel value corresponding to B22 is the value minus γ; for B21 it is the value minus double γ; and for B24 it is the value plus γ. For the three blocks in B4, B42 and B44 are the value plus γ, and B41 is the value plus double γ. The enhancement of the amplitude of the pixel value in the pixel space corresponding to different blocks thus completed, and the contrast of the individual object is thereby enhanced.

## Results

The experiment used 211 brain MRI images of ten patients set estimates according to the proposed method. The image size was 256 × 256. The brain MRI images showed WM, GM, CSF, and brain cells from the caudate nucleus, putamen, and thalamus. The experiment used the correlation histogram to successfully enhance the pathological region of PD. Then we calculated the differences in their degrees of grayscale pixel values on the gray level correlation histogram and finally calculated the variance after the image contrast was flexibly adjusted.

Common image contrast enhancement methods are HE, closing, opening, and CLAHE. The proposed method was compared to these methods. A quantitative analysis is provided in Sect. 4.1 and the results are analyzed in Sect. 4.2.

### Quantitative Analysis

Two objective evaluation methods, namely, the peak signal-to-noise ratio (PSNR) and the average gradient, were applied to compare the enhancement effects of the algorithms. The PSNR is commonly used to measure the anti-noise performance of algorithms. A higher PSNR indicates better anti-noise performance. The average gradient reflects tiny differences among pixels. A larger average gradient indicates a clearer image.

The PSNR is most easily defined with the mean squared error (MSE). Given an $M \times N$ original image $C$ and its enhanced image $S$, $MSE$ is defined as:

$$MSE = \frac{1}{M \times N}\sum\limits_{i = 1}^{M} {\sum\limits_{j = 1}^{N} {\left( {C\left( {i,j} \right) - S\left( {i,j} \right)} \right)^{2} } }$$

With $MAX_{C}$ defined as the maximum possible pixel value of the original image $C$, the PSNR is defined as:

$$PSNR = 10 \times \log_{10} \left( {\frac{{MAX_{C}^{2} }}{MSE}} \right)$$

With $S$ defined as an $M \times N$ enhanced image, the average gradient is defined as:

$$G = \frac{1}{{\left( {M - 1} \right) \times \left( {N - 1} \right)}}\sum\limits_{i = 1}^{M - 1} {\sum\limits_{j = 1}^{N - 1} {\sqrt {\frac{{\left( {S\left( {i + 1,j} \right) - S\left( {i,j} \right)} \right)^{2} + \left( {S\left( {i,j + 1} \right) - S\left( {i,j} \right)} \right)^{2} }}{2}} } }$$

Table 1 shows the quantitative analysis results for the various methods. The patient numbers are the test patients’ numbers, and the image sets are the image numbers of each patient set. When the patients’ MRI images are not clear, the image numbers of the enhanced results can be reduced by using the proposed method. HE and CLAHE adjust the contrast for the overall image and significantly reduce image quality. The opening (closing) method focuses on the dark (light) details of the image for contrast enhancement. Though the PSNR results of the two methods were better than that of the proposed method, the goal of this research was to contrast specific objects, and the overall average PSNR was better than those of the other methods. This result strongly indicates that the proposed method is suitable for contrasting specific objects.

**Table 1:** Average PSNR values for various methods

Patient no.	No. of image sets	HE	Opening	Closing	CLAHE	dB
						Proposed method
1	19	18.09	20.46	19.75	17.35	19.39
2	22	23.30	19.21	19.45	18.09	18.67
3	20	19.00	17.32	17.37	18.11	19.18
4	22	16.71	19.42	19.03	16.68	19.52
5	26	11.47	21.11	19.22	17.59	21.35
6	30	10.69	20.01	18.49	18.17	22.19
7	15	21.05	18.62	18.45	17.36	19.54
8	18	12.38	20.54	19.31	15.00	20.97
9	17	15.26	20.18	19.36	18.15	19.69
10	22	13.14	21.76	20.57	17.11	21.27
Average PSNR		16.11	19.86	19.10	17.36	20.18

Image contrast enhancement is important in the preprocessing of medical images. For example, better image enhancement can improve segmentation accuracy. This study, based on the proposed method of average gradient expression, improves the contrast of objects and enhances the edge information pertaining to objects relative to other methods and can be applied y to image segmentation research. Table 2 presents the results.

**Table 2:** Average gradient values for various methods

Patient no.	No. of image sets	HE	Opening	Closing	CLAHE	Proposed method
1	19	22.51	9.78	8.89	28.81	19.48
2	22	28.31	8.91	9.42	37.93	24.55
3	20	24.27	11.42	10.27	30.76	27.34
4	22	26.97	10.50	9.66	35.47	21.17
5	26	26.36	10.28	7.32	26.76	16.47
6	30	30.92	10.42	6.84	28.11	18.50
7	15	25.70	10.61	10.04	35.37	25.37
8	18	28.36	8.86	7.44	33.22	17.12
9	17	23.52	11.04	9.02	29.21	21.90
10	22	21.84	9.05	7.62	26.99	16.18
Average gradient		25.88	10.09	8.65	31.26	20.81

**Fig. 6:**
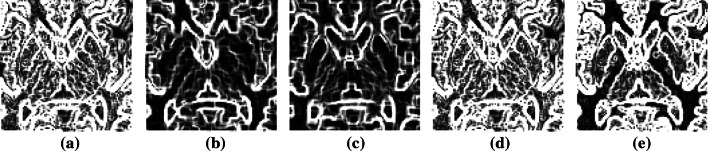
**a** HE result, **b** opening result, **c** closing result, **d** CLAHE result, and **e** proposed method of the boundary detection result in enhanced images

In Table 2, HE and CLAHE appear to be better than the proposed method because there is more edge information in the image, but there is no focus on prominent useful edges. The opening and closing methods cannot represent information related to edges. Although the result of the proposed method is inferior to those of the HE and CLAHE methods, this method can create prominent edges of specific objects. The results are significantly better than those obtained with other methods, as shown in Fig. 6.

### Comparison and Analysis

**Fig. 7:**
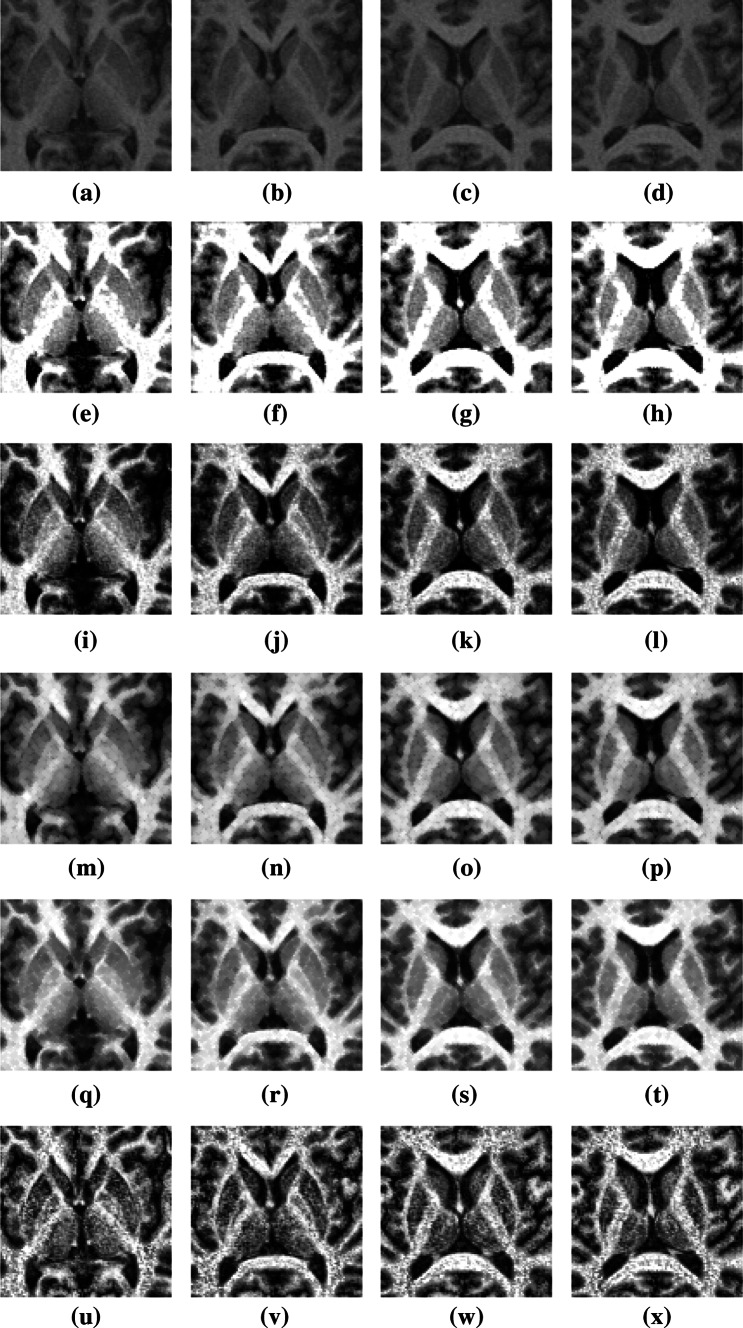
**a**–**d** MRI images of patient 1, **e**–**h** results obtained using proposed method, **i**–**l** HE images, **m**–**p** opening images, **q**–**t** closing images, and **u**–**x** CLAHE images

**Fig. 8:**
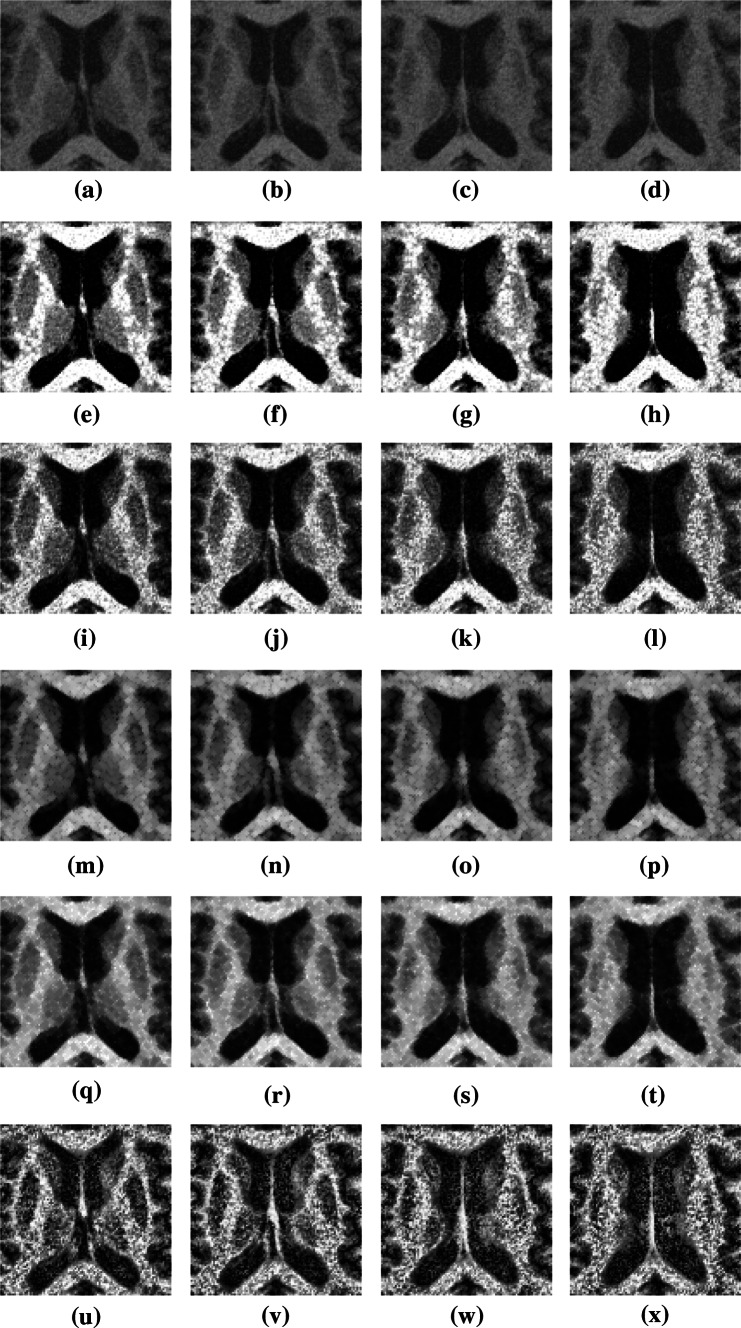
**a**–**d** MRI images of patient 2, **e**–**h** results obtained using proposed method, **i**–**l** HE images, **m**–**p** opening images, **q**–**t** closing images, and **u**–**x** CLAHE images

The proposed method was compared with the other methods. Eight test images were selected from different patients, as shown in Figs. 7 and 8. Figures 7a–d and 8a–d show four successive MRI images of the brain of patients 1 and 2, respectively. Figures 7e–x and 8e–x show the experimental results generated by the various methods. The experimental results showed that the noise was lowest for the proposed method. In addition, the proposed method has high flexibility in enhancing the region of PD-affected cells. The proposed method can automatically and correctly enhance the image contrast of brain cells in the ROI, thereby making the variation between the pixels of different cells significant. The images processed using the proposed method can be applied as references for PD diagnosis.

## Conclusion

This study proposes a method of HCHA for automatic adjustment of image contrast during the examination of three major atrophic cell areas of PD patients. When the image contrast is increased, image features become more significant. Brain cells are segmented using morphological and mathematical set operations. According to the experimental results, the contrast enhancement of brain cells obtained using the proposed method is better than that obtained with commonly used methods. This finding suggests that proposed method can be integrated with medical instruments to assist medical personnel with recognition on a CAD system, shorten the time required for manual review, and increase the efficiently of treatment.
